# Application of numerical simulation studies to determine dynamic loads acting on the human masticatory system during unilateral chewing of selected foods

**DOI:** 10.3389/fbioe.2023.993274

**Published:** 2023-05-11

**Authors:** Przemysław Stróżyk, Jacek Bałchanowski

**Affiliations:** ^1^ Faculty of Mechanical Engineering, Department of Mechanics, Materials and Biomedical Engineering, Wrocław University of Science and Technology, Wrocław, Poland; ^2^ Faculty of Mechanical Engineering, Department of Fundamentals of Machine Design and Mechatronic Systems, Wrocław University of Science and Technology, Wrocław, Poland

**Keywords:** dynamic patterns of a muscles, dynamic characteristics of foods, numerical simulation, kinematic-dynamic model of unilateral chewing, experimental tests of food

## Abstract

**Introduction:** This paper presents its kinematic-dynamic computational model (3D) used for numerical simulations of the unilateral chewing of selected foods. The model consists of two temporomandibular joints, a mandible, and mandibular elevator muscles (the masseter, medial pterygoid, and temporalis muscles). The model load is the food characteristic (*i*), in the form of the function *F_i_* = *f*(Δ*h_i_*)−force (*F_i_*) vs change in specimen height (Δ*h_i_*). Functions were developed based on experimental tests in which five food products were tested (60 specimens per product).

**Methods:** The numerical calculations aimed to determine: dynamic muscle patterns, maximum muscle force, total muscle contraction, muscle contraction corresponding to maximum force, muscle stiffness and intrinsic strength. The values of the parameters above were determined according to the mechanical properties of the food and according to the working and non-working sides.

**Results and Discussion:** Based on the numerical simulations carried out, it can be concluded that: (1) muscle force patterns and maximum muscle forces depend on the food and, in addition, the values of maximum muscle forces on the non-working side are 14% lower than on the working side, irrespective of the muscle and the food; (2) the value of total muscle contraction on the working side is 17% lower than on the non-working side; (3) total muscle contraction depends on the initial height of the food; (4) muscle stiffness and intrinsic strength depend on the texture of the food, the muscle and the side analysed, i.e., the working and non-working sides.

## 1 Introduction

Food consumption is one of the most important activities (Lund, 1991), necessary to sustain life processes at the same time as a complex kinematic-dynamic process (Daumas et al., 2005; Hedjazi et al., 2013; Peck et al., 2000; Piancino et al., 2008; Slager et al., 1997; Stróżyk and Balchanowski, 2018), which is controlled by the central nervous system (Dellow and Lund, 1971; Lund, 1991). Mastication is also a multi-parametric issue encompassing many complex and synchronised processes for preparing a bolus for swallowing. The most important processes may include: 1) the dynamic processing of the food (Stokes et al., 2013) dependent on its position on the dental arch (Manns and Díaz, 1988), 2) the continuous change in the geometric dimensions and mechanical properties of the food (Lenfant et al., 2009; Lillford, 2000; Lucas et al., 2004; Mioche et al., 2002; Stokes et al., 2013) and 3) the performance of complex movements by the mandible during chewing (Hylander, 2006; Koolstra, 2002; Piancino et al., 2012; Posselt, 1952; Quintero et al., 2013; Slavicek, 2010; Weinberg, 1963).

There are many papers in the literature where the authors (Çakir et al., 2011; Hutchings et al., 2012; Kohyama et al., 2007; Kohyama et al., 2008; Santana-Mora et al., 2014; Wang et al., 2010) focus on various parameters related to the act of chewing, e.g., 1) change in food texture, 2) chewing effort, 3) muscle activity, 4) some cycles needed to prepare a bolus of food, 5) degree of wetting of food with saliva, 6) age, 7) pathogenic changes, but most importantly 8) loads that occur during chewing of food. On the other hand, based on the data reported by (Agrawal et al., 1998; Hiiemae et al., 1996; Koolstra, 2002; Mathevon et al., 1995; Shimada et al., 2012), it appears that the mechanical properties of food are important for the function of the masticatory system during symmetric incisal biting and chewing. Furthermore, the work results (Mioche et al., 1999; Stróżyk and Bałchanowski, 2018) indicate that food imposes individual patterns of muscular force that must adapt to different functional requirements (Fitts et al., 1991). Their values are controlled by the central nervous system (Dellow and Lund, 1971; Lund, 1991), based on remembered external stimuli (patterns) perceived by different receptors (Quintero et al., 2013) so that it is adapted to the food consumed (Hiiemae et al., 1996; Knudson, 2007; Murray, 2004).

One of the main ways to determine muscle forces is through electromyography (EMG)-based method and mathematical equations (Ferrario and Sforza, 1996; Itoh et al., 1997; Mioche et al., 1999; Murray et al., 1999; Pruim et al., 1980; Weijs and Hillen, 1985). The method is mainly applied to the masseter muscle and the temporalis muscle. In the case of the medial and lateral pterygoid muscles, measurements are more difficult because they cannot be externally accessed (Koole et al., 1990; Murray et al., 1999; Widmalm et al., 1987; Wood et al., 1986). Consequently, determining muscle forces requires intra-oral access, which makes measurements during chewing difficult due to the possibility of interference with the natural act of chewing.

Since using EMG during chewing makes it impossible to determine the forces in all the mandibular elevator muscles, it is a good option to use a numerical computational model of the masticatory system. The model developed for numerical calculations provides reproducibility but requires the preparation of basic data, e.g., the geometry of the mandible, the muscle attachment sites, the muscle model and the mode of support. As chewing is a dynamic issue, a model should be developed based on solid mechanics, i.e., a kinematic-dynamic model (Stróżyk and Bałchanowski, 2016; Stróżyk and Bałchanowski, 2018; Stróżyk and Bałchanowski, 2020). The load of the model should be the characteristics of the food in the form of the function *F*_*i*_ = *f*(Δ*h*_*i*_)—force (*F*_*i*_) vs. change in specimen height (Δ*h*_*i*_), determined during experimental tests, while the movement of the mandible is set in the path of mastication determined for the tested food.

Preliminary analysis of the function of the masticatory system in terms of: 1) the position of the food (bite force) on the dental arch, 2) the number of cycles and 3) the muscle activity on the left and right sides of the mandible indicate that, in mechanical terms, unilateral chewing will be an interesting case of physiological load on the masticatory system.

In the literature, it is possible to find publications in which the authors model unilateral chewing, but these are static models in which the position of the mandible corresponds to the closure of the mouth (Korioth et al., 1992; Pinheiro and Alves, 2015; Pachnicz and Stróżyk, 2021; Reina et al., 2007). Interesting information on unilateral chewing can be found in (Harrison et al., 2014). However, the authors focus on dynamic changes in the mechanical properties of the food rather than on changes in the values of the muscular forces acting on the masticatory system. Analysing other publications on chewing (Choi et al., 2005; Jahadakbar et al., 2016; Lee et al., 2017; Luo et al., 2017; Marková and Gallo, 2016; Reina et al., 2007), we find that none of them present changes in muscle force values (muscle functioning patterns) to dynamic changes occurring in the food during mastication.

The primary aim of this study was to determine computational model of unilateral chewing, loaded with the dynamic patterns of food (i) determined in experimental tests. The model prepared in this way made it possible to determine, firstly, selected dynamic parameters of the mandibular elevator muscles, i.e., total muscle contraction (*q*_*i*_) and dynamic muscle forces (*F*_*i*_) as functions, *q*_*i*_ = *qi*(*t*) and *F*_*i*_ = *F*_*i*_(*t*), respectively. The parameters obtained allowed the determination of muscle dynamic patterns in the function *F*_*i*_ = *f*(*q*_*i*_). From the results obtained, parameters such as muscle stiffness (*K*_*i*_) and intrinsic strength (*k*_*i*_) were also determined as a function of food.

As mastication is a complex mechanical process (Stokes et al., 2013), the study was limited to first cycle.

The results demonstrate the feasibility of using a hybrid model (based on experimental tests of food and numerical simulation of unilateral chewing) to show how the dynamic parameters of the mastication system change as a function of the mechanical properties of the food and its geometric dimensions.

The proposed computational model, the adopted boundary conditions and the calculation method can be one possibility for determining muscular forces. On the other hand, the results obtained can be used to model complex issues concerning the biomechanics of the masticatory system requiring knowledge of loads.

## 2 Materials and methods

The use of numerical simulation to determine force patterns for the mandibular elevator muscles (the masseter muscle-M, the medial pterygoid muscle-MP and the temporalis muscle-T) during unilateral chewing required, first of all, the construction of a numerical model of the human masticatory system consisting of the mandible, TMJ and muscles. In addition, this necessitated: 1) determining how to support the model and 2) defining and determining the parameters (input data) responsible for the load and initial position (for time *t =* 0*s*) of the model.

### 2.1 Determination of model load and food characteristics

During the numerical simulations, forces in the muscles were determined for loads corresponding to different foods; therefore the basic parameter responsible for the mandibular force is the food characteristic (*i*), in the form of the function *F*_*i*_ *=f*(Δ*h*_*i*_)—force (*F*_*i*_) vs. change in high of food specimen (Δ*h*_*i*_), determined in experimental studies. In the numerical model, the above function is decomposed into two functions in which force and change in high of food specimen are time-dependent, i.e., *F*_*i*_(*t*) and *Δh*_*i*_(*t*).

The general algorithm for determining food characteristics was similar to that presented in (Stróżyk and Bałchanowski 2018). Five foods were prepared for the study (*i = c, a, d, b, s*): 1) vegetables—carrot (*c*); 2) fruits—apple (*a*); 3) sweets—dark chocolate (*d*) and a chocolate bar (*b*) and 4) meat and cold cuts—sausage (s), different in terms of structure, mechanical properties and method of production (natural and artificial). The products were purchased from a single selected grocery shop and stored at 7°C until the specimens were prepared. The specimens were then normalised at 21°C for approximately 60 min until the tests began.

The aim of the experimental study was not to determine typical mechanical parameters but only the function *F*_*i*_ *=f*(Δ*h*_*i*_). The specimens in terms of dimensions [height (*h*_*i*_), width (*w*_*i*_). and length (*l*_*i*_)] were similar to a typical bite of food, while the shape depended on the product (Table 1). To determine the characteristics, 60 specimens were prepared for each food.

**TABLE 1:** Food product (*i*), its mean height (*h*_*i*_), width (*w*_*i*_) and length (*l*_*i*_) and mean chewing time (*t*_*i*_).

Parameters	Dark chocolate (*d*)	Chocolate bar (*b*)	Apple (*a*)	Carrots (*c*)		Sausage (*s*)	
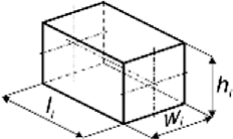	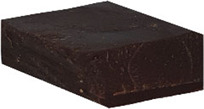	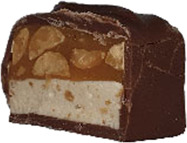	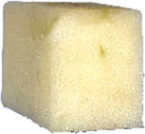	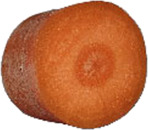		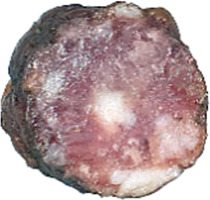	
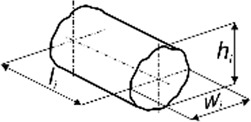							
*h*_*i*_ /^1^ [×10^−3^ m]	9.1 ± 0.8	20.1 ± 0.4	23.5 ± 1.2	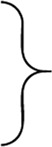	19.6 ± 1.5	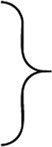	27.2 ± 1.8
*w*_*i*_ /^1^ [×10^−3^ m]	17.1 ± 1.2	17.4 ± 1.4	18.6 ± 0.8				
*l*_*i*_ /^1^ [×10^−3^ m]	26.2 ± 0.8	30.3 ± 0.7	26.5 ± 0.6		16.8 ± 1.1		17.4 ± 1.3
*t*_*i*_ /^1^ [s]	0.51 ± 0.03	1.12 ± 0.07	1.31 ± 0.09	1.08 ± 0.08		1.51 ± 0.09	

^/1^ the values are means ± SD, (*i* = *d, b, a, c, s*).

**FIGURE 1:**
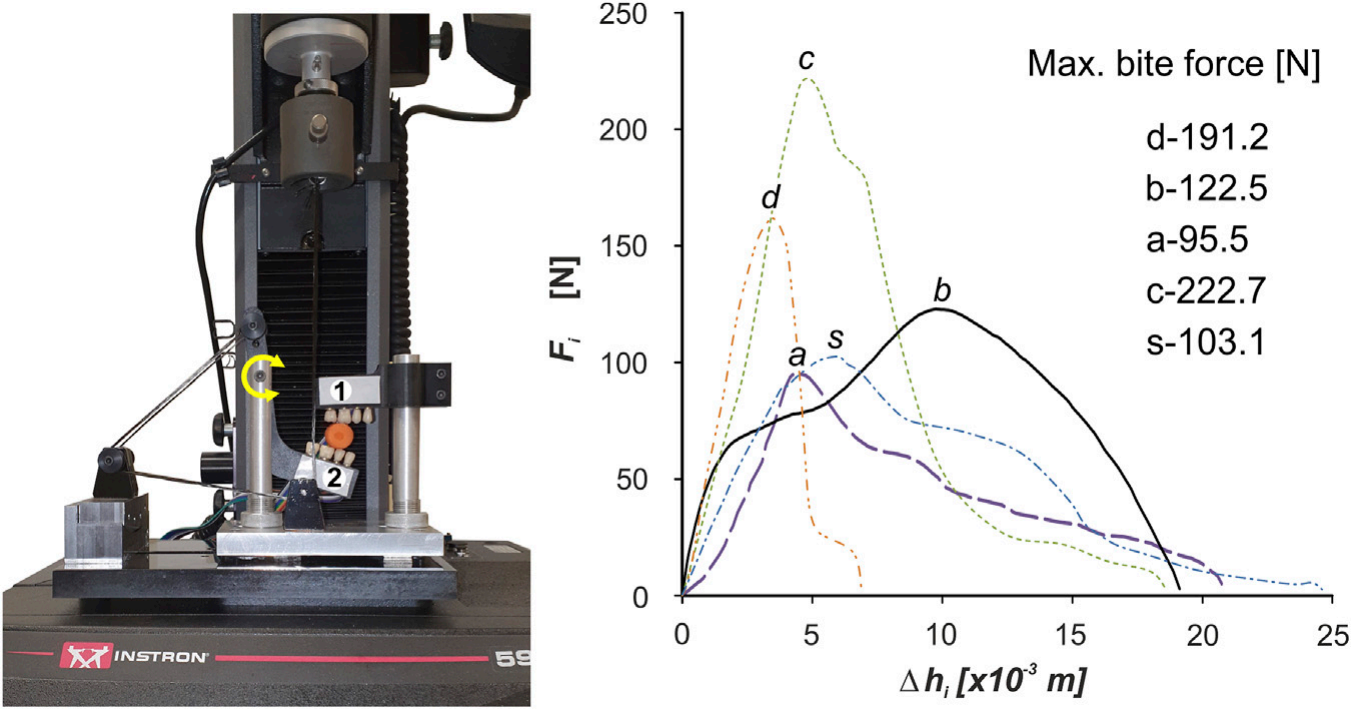
Unilateral chewing measuring system used in the experimental study to determine the characteristics of food products (*i*) in the form of the function *F*_*i*_ *=f*(Δ*h*_*i*_) (*i* = dark chocolate (*d*), chocolate bar (*b*), apple (*a*), carrots (*c*) and sausage (*s*)]; 1–upper head (maxilla), 2–lower head (mandible).

In the experimental tests, we used our own test stand design (Figure 1) [developed on the basis of a patent application—(Stróżyk, 2021)], consisting of two measuring heads, i.e., upper (maxilla) and lower (mandible), imitating fragments of the dental arches.

The lower head is mounted on a cantilever and thus can perform a rotational. In addition, acrylic dental prostheses, used in dental prosthetics, were attached to the heads to make the act of chewing similar to natural chewing. During testing, the test stand was mounted on an Instron 5944 test machine.

Based on the information provided in (Fitts et al., 1991; Dick and Wakeling, 2017; Koolstra, 2002), muscle force is dependent on the speed of mandibular movement, while speed is dependent on the mechanical parameters of the food, i.e., high force and low speed (hard food) or low force and high speed (soft food). Furthermore, based on the analysis of (Anderson et al., 2002; Foegeding and Drake, 2007; Foster et al., 2006; Meullenet et al., 2002; Williams et al., 2005) it appeared that the range of reported mandibular movement speeds (in this case, chewing speed) for different foods is extensive. Therefore, it was decided that all foods would be masticated at a constant velocity of *v*_*t*_ = 0.02 m/s (Stróżyk and Bałchanowski, 2016; Stróżyk and Bałchanowski, 2018; Stróżyk and Bałchanowski, 2020). Maintaining a constant *v*_*t*_ required performing a kinematic analysis of the simulator and, based on this, developing a control program for the Instron 5944 machine (Bluehill ver. 3). In addition, the chewing time (*t*_*i*_) was determined for each food product based on the nominal food height *h*_*i*_) and the set velocity *v*_*t*_ (Table 1).

Figure 1 shows the characteristics of selected products (*i*), in the form of the function *F*_*i*_ *= f*(Δ*h*_*i*_), developed on the basis of measurement results and elementary statistical calculations.

### 2.2 Numerical model of the human masticatory system

In order to carry out a kinetostatic analysis of the chewing process of selected foods, a computational model of the human masticatory system was developed, consisting of two elements: a fixed skull (Synbone 8500) and a movable mandible (Synbone 8596). The temporomandibular joints (TMJ) were modelled as shaped joints, with constraints in the form of contact forces between the articular surface of the condyle of the mandible and the articular tuber of the temporal bone (Strozyk and Balchanowski, 2018). The forces in the muscles were modelled using non-linear force vectors applied at the anatomical points of muscle attachment.

**FIGURE 2:**
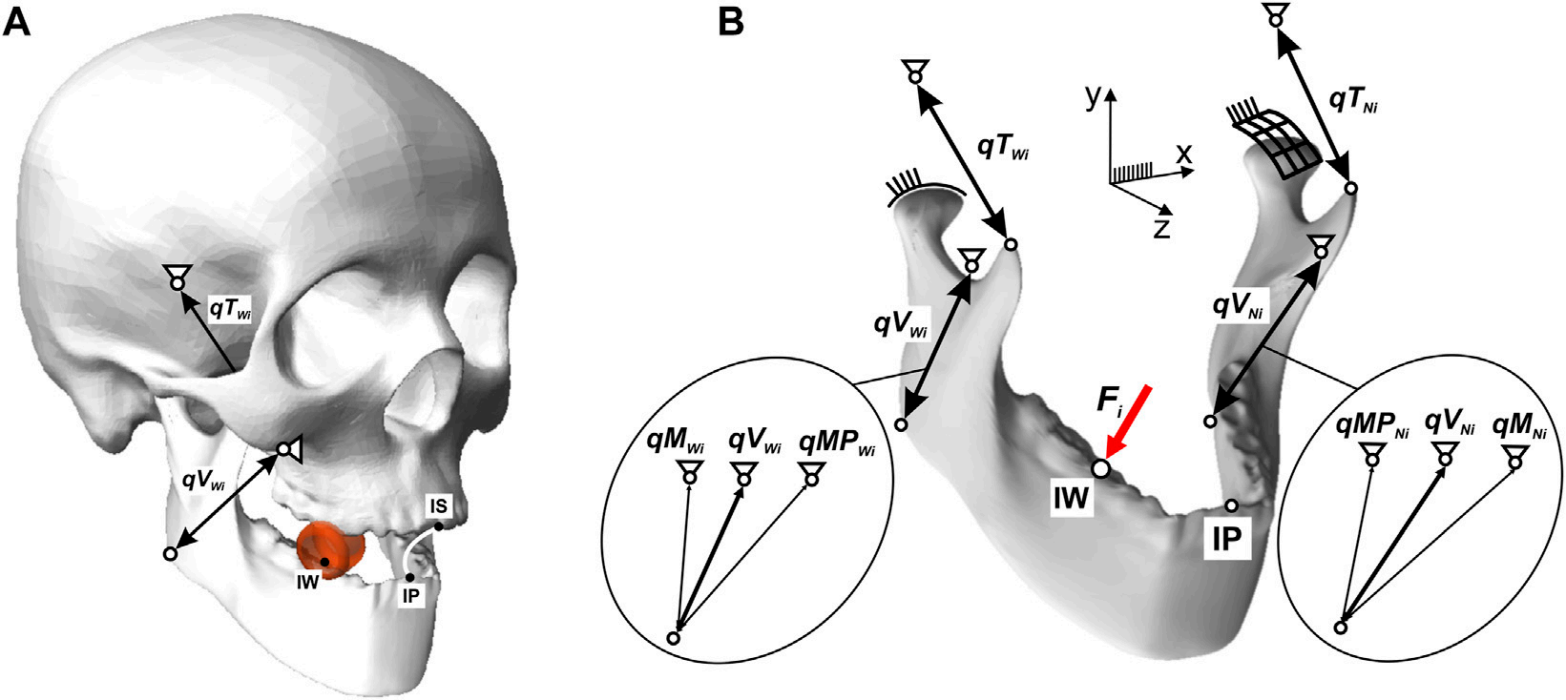
Computational model of the human masticatory system: **(A)** general view, **(B)** force forcing diagram of the mandible.

The numerical model had 4 degrees of freedom, which means that 4 active muscle force unknowns can be determined from the equilibrium equations. Hence, the model assumed the unknowns were the two temporalis muscle forces (*FT*_*Wi*_, *FT*_*Ni*_). In contrast, the forces in the masseter muscle (*FM*_*Wi*_, *FM*_*Ni*_) and the medial pterygoid muscles (*FMP*_*Wi*_, *FMP*_*Ni*_) were replaced by the resultant forces (*FV*_*Wi*_, *FV*_*Ni*_) (Prium et al., 1980), on the left and right sides (Figure 2).

The muscles were modelled using linear kinematic excursions to simulate the elongation and contraction of the muscles that allow the mandible to move in relation to the maxilla during mastication. In the numerical model of the skull−mandible, four linear kinematic excursions were used for the temporalis muscle and the resultant. The active forces *FT*_*Wi*_, *FT*_*Ni*_, in the enforcing *qT*_*Wi*_, *qT*_*Ni*_ represent the temporalis muscle, while the active forces *FV*_*Wi*_, *FV*_*Ni*_ in the enforcing *qV*_*Wi*_ and *qV*_*Ni*_ represent the results of the masseter and medial pterygoid muscles (Figure 2).

The external force on the model [modelling the occlusal force (Stróżyk et al., 2018; Stróżyk and Bałchanowski, 2016; Stróżyk and Bałchanowski, 2018)] was the force *F*_*i*_, as a function of *F*_*i*_ *= f*(Δ*h*_*i*_), applied at point *IW* on the occlusal surface of the first molar (46) (Figure 2).

### 2.3 Boundary conditions for the numerical model

Based on an analysis based on solid mechanics and analytical geometry, it appears that the parameters that will have a significant effect on the alignment of the mandible with respect to the maxilla will be: 1) product height (*h*_*i*_) and 2) unilateral contraction of the lateral pterygoid muscle. The height of *h*_*i*_ is responsible for establishing the distance between the upper and lower incisors, whereas contraction of the muscle on the non-working side (*N*) is responsible for lateral movement of the mandible, resulting in the appearance of a path of mastication and asymmetrical displacement of the processes in the TMJ during unilateral chewing. Since there is a lack of data in the available literature on the preload (muscle force) and corresponding stiffness of the lateral pterygoid muscle, it was decided to use the effect of its action as a parameter defining mandibular preposition and movement.

The appropriate association of the height of the *h*_*i*_ and the mastication path (effect) and the appropriate alignment of the mandibular processes at the TMJ enable the initial position of the model to be determined during unilateral chewing.

**FIGURE 3:**
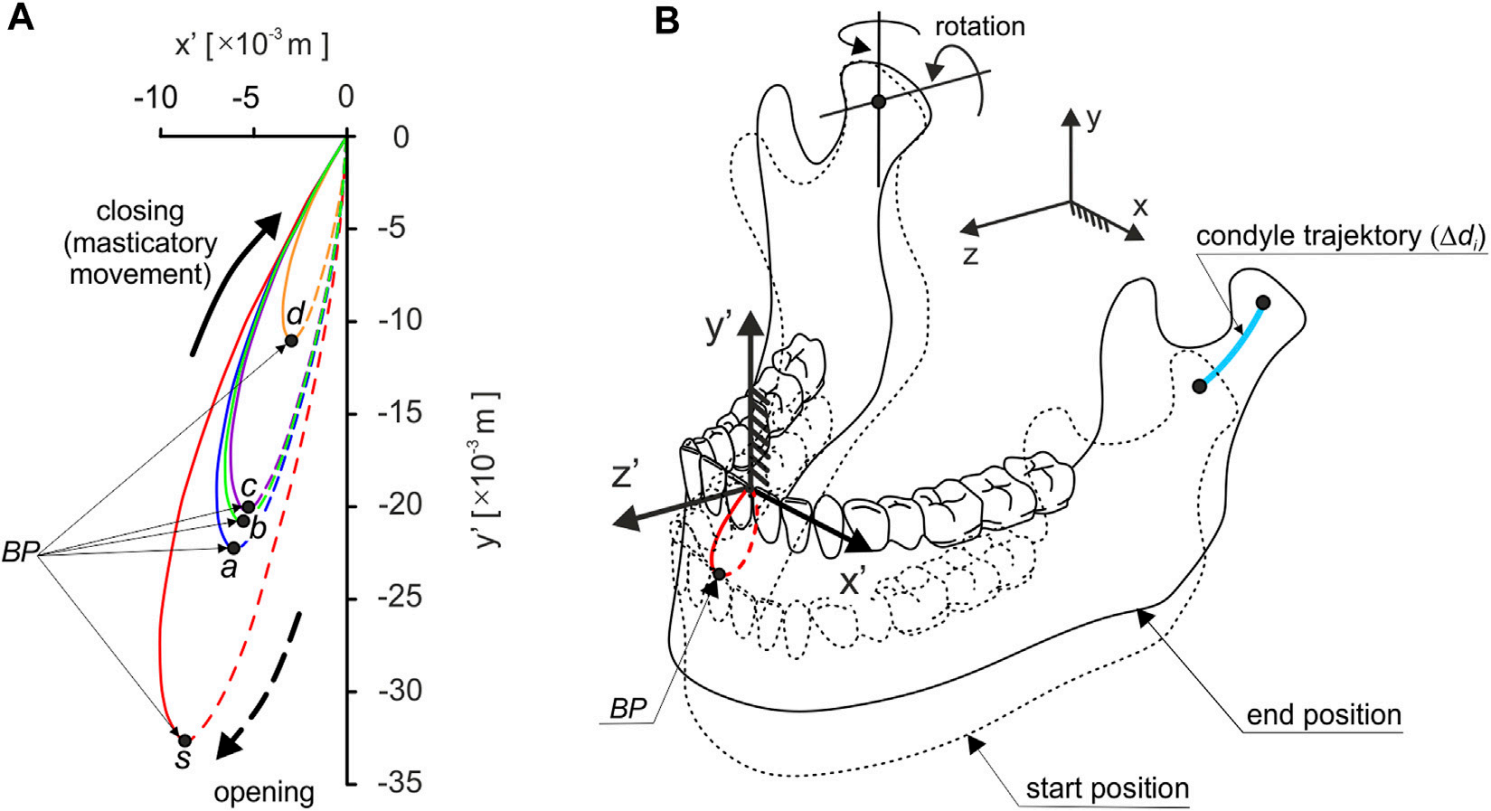
Trajectory of: **(A)** the incisal point in the frontal plane (path of chewing), depending on the foods and **(B)** the mandibular condyle on the non-working side during unilateral chewing; Black point (*BP*) indicates the point at which chewing begins.

Based on the analysis of data reported in (Bhatka et al., 2004; Buschang et al., 2007; Nishigawa et al., 1997; Piancino et al., 2012; Slavicek, 2010), a hypothetical path of mastication was prepared for each product, in the frontal plane (Figure 3A), in shape close to an ellipse (Hedjazi et al., 2013), in such a way that its geometric parameters (height and width) were synchronised with the food height (*h*_*i*_), i.e., the initial distance between a pair of corresponding first molars.

The path of mastication prepared in this way are the parameter by which it is possible to reproduce the trajectory of the incisal point during a single or one cycle of unilateral chewing.

The results of preliminary calculations showed that the introduction of the mastication path significantly affects the values of the length of the condylar trajectories (Δ*d*_*i*_). In addition, analysis of the results showed that the condyles move according to the mandibular kinematics reported in (Weinberg, 1963), i.e., the working condylar rotates and little lateral Bennett’s movement. In contrast, the non-working condylar movement is sloped downward, forward, and medially.

For the non-working side, the length of the trajectory of the condylar is: chocolate Δ*d*_*d*_ = 0.0031 m, chocolate bar Δ*d*_*b*_ = 0.0068 m, apple Δ*d*_*a*_ = 0.0079 m, carrot Δ*d*_*c*_ = 0.0065 m, and sausage Δ*d*_*s*_ = 0.0102 m, and is on average as much as seven times greater than on the working side. Therefore, in the numerical simulation, it was assumed that there would be a fixed centre of rotation of the mandible in the condylar on the working side, through which the instantaneous axes of rotation would pass. On the non-working side, on the other hand, the condylar will have the possibility of rotation and translation (Figure 3B).

The mandibular model is fixed in the TMJ and the origin attachment sites of the masseter, medial pterygoid, and temporalis muscles. Based on the developed chewing paths, there are kinematic contact pairs with 5 degrees of freedom (3 rotations and 2 displacements) in the TMJ on the working and non-working sides. The displacement values depend on the type of food and the chewing side (Table 1).

The proposed way of supporting the model, especially in the TMJ, made it possible to simulate the complex movement of the lower incisors during chewing (Figures 2, 3). A detailed description of the model is given in (Stróżyk and Bałchanowski, 2018).

### 2.4 Numerical simulations of unilateral chewing

Simulation studies consisted in moving the incisal point from the lower position (point IP Figure 2A) to the endpoint IS (the origin of the coordinate system Figure 2).

During the simulation of food chewing, the molar to which the force *F*_i_ has applied moves with a constant velocity *v*_t_ = 0.02 m/s. The developed numerical model is universal and can model variable (nonlinear) chewing velocity. In the simulation studies chewing was modelled at a constant speed because the food patterns were determined experimentally on a testing machine for such speed.

**FIGURE 4:**
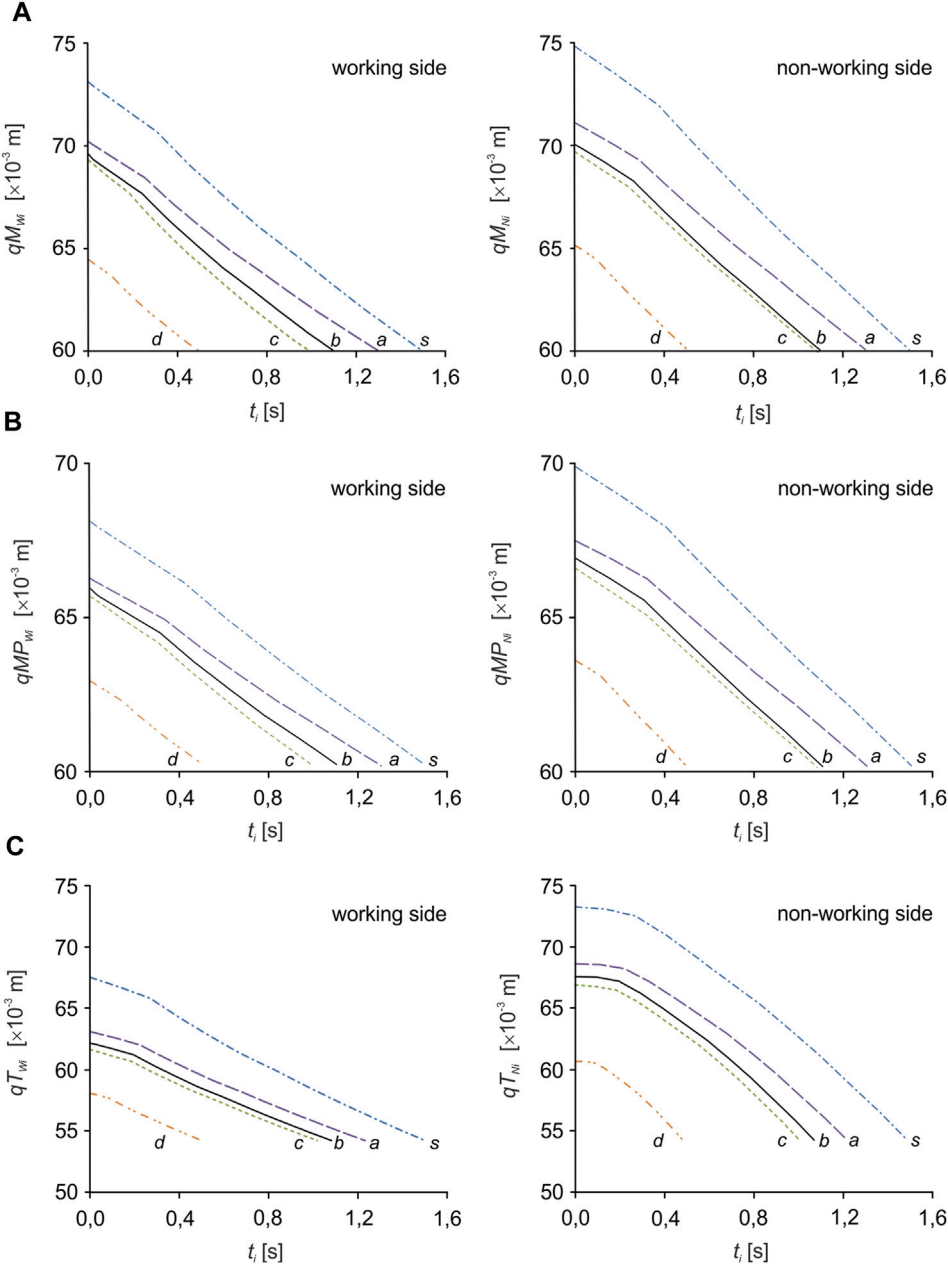
Kinematic characteristics of the change in muscle length: **(A)** the masseter muscle (*qM*_*Wi*_, *qM*_*Ni*_), **(B)** the medial pterygoid muscle (*qMP*_*Wi*_, *qMP*_*Ni*_) and **(C)** the temporalis muscle (*qT*_*Wi*_, *qT*_*Ni*_) depending on the food product (*i*).

Numerical simulations were carried out in three stages. In the first, the inverse task of the kinematics of motion of the mandibular-cranial system was solved. This consisted in applying a forced mandibular movement along the longitudinal mastication path and determining the mandibular movement at the TMJ on the W and N sides. During this simulation, the changes in muscle length (contractions) necessary to force the mandibular closure movement were also determined (Figure 4).

In the second stage, proper calculations were performed modelling a simple dynamics task simulating mandibular closure resulting from muscle contractions. Forcing *qT*_*Wi*_, *qT*_*Ni*_, *qV*_*Wi*_ and *qV*_*Ni*_ were used as changes in muscle length (contractions) determined in the simple task. As a result of calculations, the unknown muscle forces were determined: *FM*_*Wi*_, *FM*_*Ni*_, *FMP*_*Wi*_, *FMP*_*Ni*_ and *FT*_*Wi*_, *FT*_*Ni*_. Simulations were performed separately for each food.

**FIGURE 5:**
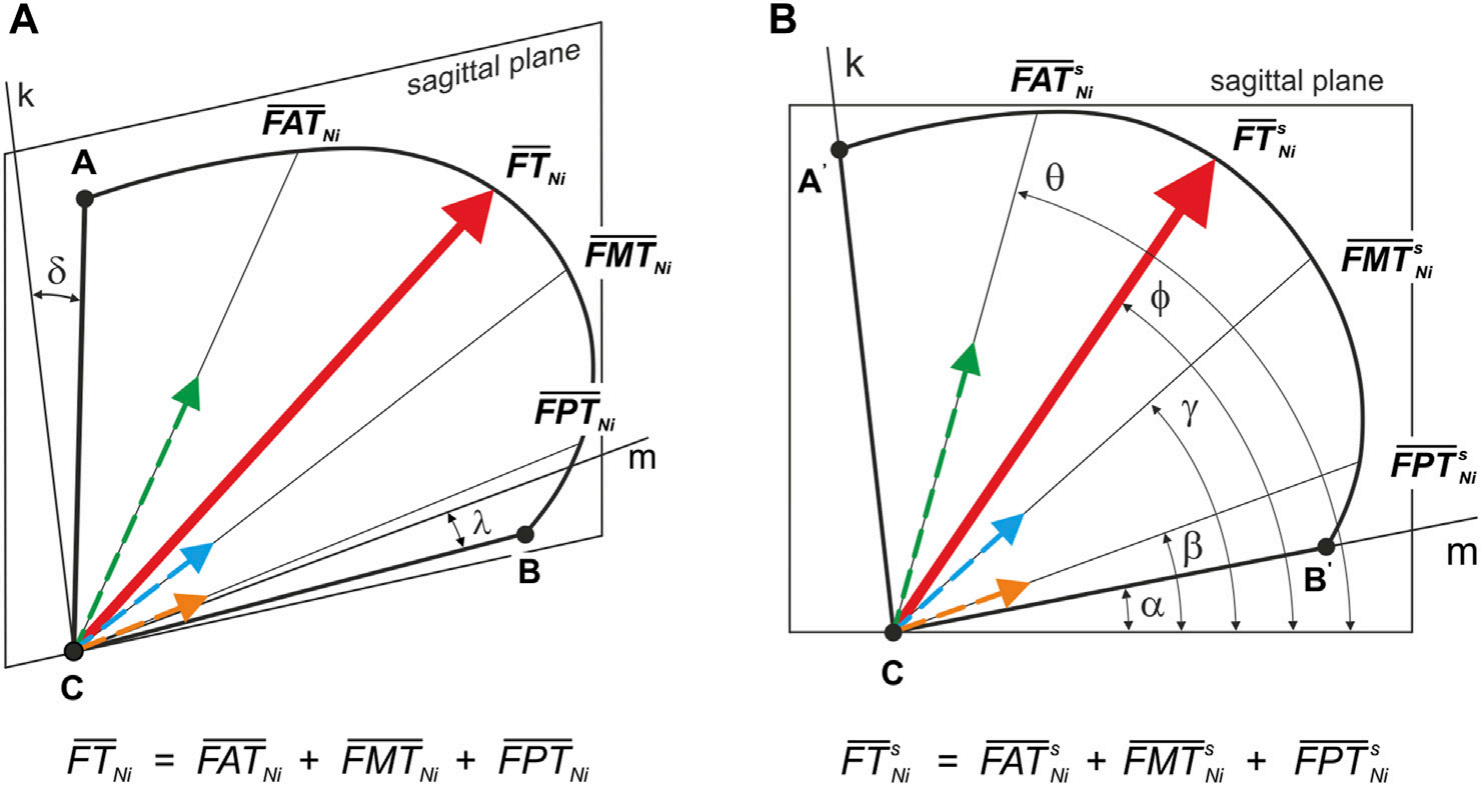
Position of the principal vector of the temporalis muscle and its components–working and non-working side: **(A)** relative to the sagittal plane ( $\bar{{FT}_{Ni}}$ ; $\bar{{FAT}_{Ni}}$ ; $\bar{{FMT}_{Ni}}$ ; $\bar{{FPT}_{Ni}}$ ) and **(B)** on the sagittal plane ( $\bar{{FT}_{Ni}^{s}}$ ; $\bar{{FAT}_{Ni}^{s}}$ ; $\bar{{FMT}_{Ni}^{s}}$ ; $\bar{{FPT}_{Ni}^{s}}$ ). (*δ = 20° i λ = 20°*); Line k and m lie in the sagittal plane.

In the third step, based on elementary trigonometric calculations, the principal vector of the temporalis muscle was decomposed (*FT*_*Ni*_) into three components, respectively for: the anterior temporalis (*FAT*_*Ni*_), the middle temporalis (*FMT*_*Ni*_) and the posterior temporalis (*FPT*_*Ni*_). The distribution was performed based on the assumption that muscle force is proportional to physiological cross-sectional area (PCSA) (Koolstra et al., 1988); Figure 5A shows the schematic position of the temporalis muscle (outline—ABC) in relation to the sagittal plane. On the other hand, Figure 5B shows the position of the temporalis muscle (outline—A'B'C) in the sagittal plane and gives the angles determining the position of the B'C edge (*α* = 11°) and the line of action of *FT*_*Ni*_ (*ϕ* = 58°) and its components, i.e., *FAT*_*Ni*_ (*θ* = 76°), *FMT*_*Ni*_ (*γ* = 42°) and *FPT*_*Ni*_ (*β* = 22°), in relation to the sagittal axes.

## 3 Results

**FIGURE 6:**
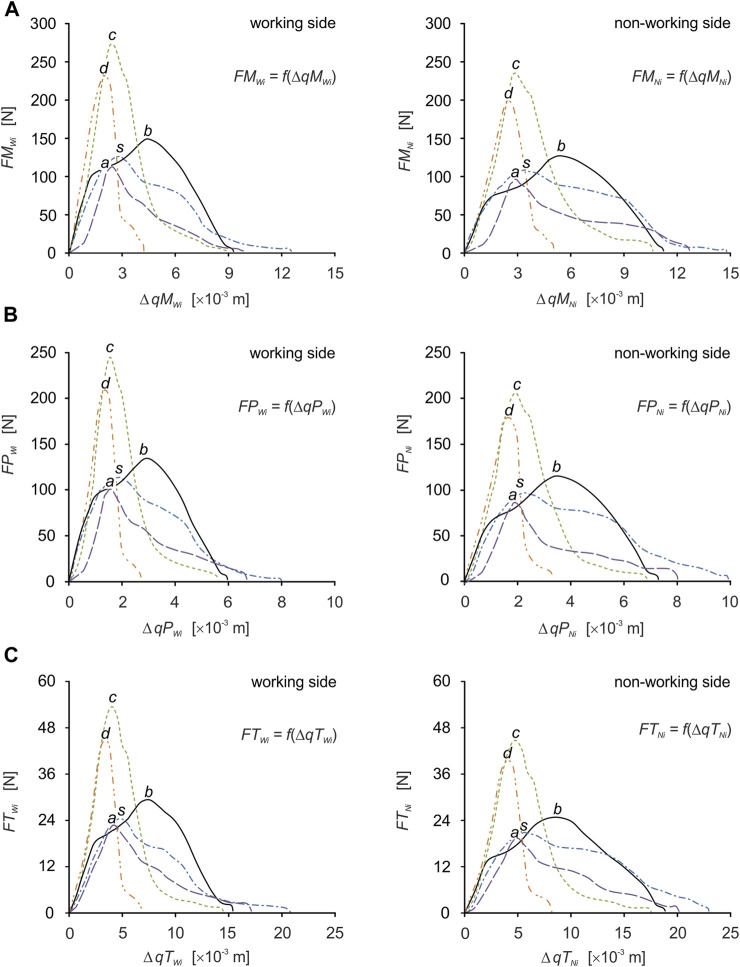
Muscle force patterns as a function of muscle force vs. muscle contraction for: **(A)** the masseter muscle [*FM*_*Wi*_ *= f*(*ΔqM*_*Wi*_), *FM*_*Ni*_ *= f*(*ΔqM*_*Ni*_)], **(B)** the medial pterygoid muscle [*FMP*_*Wi*_ = *f*(*ΔqMP*_*Wi*_), *FP*_*Ni*_ = *f*(*ΔqMP*_*Ni*_)] and **(C)** the temporalis muscle [*FT*_*Wi*_ = *f*(*ΔqT*_*Wi*_), *FT*_*Ni*_ *= f*(*ΔqT*_*Ni*_)], during unilateral food chewing (*i*).

Based on the determined food characteristics (Figure 1), the developed model of the masticatory system (Figure 2), the chewing path (Figure 3), the determined changes in muscle length (Figure 4) and numerical calculations, muscle force patterns were determined, for the masseter muscle, the medial pterygoid muscle and the temporalis muscle and its components (the anterior temporalis, the middle temporalis, the posterior temporalis) (Figure 6). In addition, Tables 2, 3 give the values for maximum bite force and the corresponding values for muscle contraction and total muscle contraction. In contrast, Tables 4, 5 show muscle stiffness and intrinsic strength values.

**TABLE 2:** Maximum muscle force, contraction corresponding to maximum force and total contraction, respectively for: the masseter muscle [(*FM*_*HWi*_, *FM*_*HNi*_), (*ΔqM*_*HWi*_, *ΔqM*_*HNi*_), (*ΔqM*_*CWi*_, *ΔqM*_*CNi*_)], the medial pterygoid muscle [(*FMP*_*HWi*_, *FMP*_*HNi*_), (*ΔqMP*_*HWi*_, *ΔqMP*_*HNi*_), (*ΔqMP*_*CWi*_, *ΔqMP*_*CNi*_)] and the temporalis muscle [(*FT*_*HWi*_, *FT*_*HNi*_), (*ΔqT*_*HWi*_, *ΔqT*_*HNi*_), (*ΔqT*_*CWi*_, *ΔqT*_*CNi*_)], depending on a product (*i*).

Side	Parametry		Dark chocolate (d)	Chocolate bar (b)	Apple (a)	Carrots (c)	Sausage (s)
Masseter							
Working	*FM*_*HWi*_	[N]	233.2	149.4	112.2	274.3	126.9
	*ΔqM*_*HWi*_	[×10^−3^ m]	2.1	4.4	2.4	2.4	2.9
	*ΔqM*_*CWi*_	[×10^−3^ m]	4.2	9.3	10.5	8.9	12.5
Non-working	*FM*_*HNi*_	[N]	200.3	127.8	96.2	235.5	108.8
	*ΔqM*_*HNi*_	[×10^−3^ m]	2.5	5.3	2.9	2.8	3.5
	*ΔqM*_*CNi*_	[×10^−3^ m]	5.0	11.2	12.7	10.6	14.9
Medial pterygoid							
Working	*FP*_*HWi*_	[N]	209.3	134.5	100.6	244.7	113.5
	*ΔqP*_*HWi*_	[×10^−3^ m]	1.4	2.8	1.5	1.5	1.9
	*ΔqP*_*CWi*_	[×10^−3^ m]	2.7	5.9	6.7	5.6	8.0
Non-working	*FP*_*HNi*_	[N]	179.2	115.4	86.2	209.8	97.3
	*ΔqP*_*HNi*_	[×10^−3^ m]	1.7	3.5	1.9	1.9	2.3
	*ΔqP*_*CNi*_	[×10^−3^ m]	3.3	7.3	8.3	6.9	9.8
Temporalis							
Working	*FT*_*HWi*_	[N]	46.1	29.3	22.7	53.4	24.4
	*ΔqT*_*HWi*_	[×10^−3^ m]	3.4	7.3	3.9	3.0	4.8
	*ΔqT*_*CWi*_	[×10^−3^ m]	6.7	15.4	16.9	14.5	20.6
Non-working	*FT*_*HNi*_	[N]	39.4	25.0	19.4	45.9	20.9
	*ΔqT*_*HNi*_	[×10^−3^ m]	4.2	8.9	4.7	4.7	5.8
	*ΔqT*_*CNi*_	[×10^−3^ m]	8.2	18.8	20.4	17.5	24.9

**TABLE 3:** Maximum muscle force, contraction corresponding to maximum force and total contraction respectively for: the anterior temporalis [(*FAT*_*HWi*_, *FAT*_*HNi*_), (*ΔqAT*_*HWi*_, *ΔqAT*_*HNi*_), (*ΔqAT*_*CWi*_, *ΔqAT*_*CNi*_)], the middle temporalis [(*FMT*_*HWi*_, *FMT*_*HNi*_), (*ΔqMT*_*HWi*_, *ΔqMT*_*HNi*_), (*ΔqMT*_*CWi*_, *ΔqMT*_*CNi*_)] and the posterior temporalis [(*FPT*_*HWi*_, *FPT*_*HNi*_), (*ΔqPT*_*HWi*_, *ΔqPT*_*HNi*_), (*ΔqPT*_*CWi*_, *ΔqPT*_*CNi*_)] depending on a product (*i*).

Side	Parametry		Dark chocolate (d)	Chocolate bar (b)	Apple (a)	Carrots (c)	Sausage (s)
Anterior temporal							
Working	*FAT*_*HWi*_	[N]	22.1	14.1	10.9	25.7	11.7
	*ΔqAT*_*HWi*_	[×10^−3^ m]	2.7	5.8	3.2	3.3	3.8
	*ΔqAT*_*CWi*_	[×10^−3^ m]	5.4	12.3	13.9	12.3	16.3
Non-working	*FAT*_*HNi*_	[N]	18.9	12.0	9.3	22.0	10.0
	*ΔqAT*_*HNi*_	[×10^−3^ m]	3.4	7.2	3.9	3.8	4.6
	*ΔqAT*_*CNi*_	[×10^−3^ m]	6.6	15.1	16.9	14.5	19.7
Middle temporal							
Working	*FMT*_*HWi*_	[N]	13.4	8.5	6.6	15.5	7.1
	*ΔqMT*_*HWi*_	[×10^−3^ m]	2.5	5.2	2.9	3.0	3.4
	*ΔqMT*_*CWi*_	[×10^−3^ m]	4.7	11.0	12.2	11.0	14.4
Non-working	*FMT*_*HNi*_	[N]	11.4	7.2	5.6	13.3	6.1
	*ΔqMT*_*HNi*_	[×10^−3^ m]	2.9	6.3	3.3	3.3	4.0
	*ΔqMT*_*CNi*_	[×10^−3^ m]	5.7	13.5	14.7	12.7	17.5
Posterior temporal							
Working	*FPT*_*HWi*_	[N]	10.6	6.7	5.2	12.3	5.6
	*ΔqPT*_*HWi*_	[×10^−3^ m]	2.1	4.4	2.4	2.5	3.0
	*ΔqPT*_*CWi*_	[×10^−3^ m]	4.1	9.2	10.4	9.3	12.9
Non-working	*FPT*_*HNi*_	[N]	9.1	5.7	4.5	10.6	4.8
	*ΔqPT*_*HNi*_	[×10^−3^ m]	2.5	5.4	2.9	2.9	3.5
	*ΔqPT*_*CNi*_	[×10^−3^ m]	4.9	11.4	12.6	10.8	15.0

**TABLE 4:** Values of stiffness (*K*_*Wi*_, *K*_*Ni*_) for selected muscles in relation to food.

Side	Muscle	Chocolate (d)	Chocolate bar (b)	Apple (a)	Carrots (c)	Sausage (s)
*K*_*Wi*_ [×10^3^ N/m]						
Working	Masseter	108,9	34,0	46,3	114,8	43,5
	Medial pterygoid	152,1	48,2	65,1	162,7	60,8
	Temporalis	13,6	4,0	5,8	13,7	5,1
	Anterior temporalis	10,3	3,2	4,5	10,7	4,0
	Middle temporalis	6,2	1,9	2,7	6,5	2,4
	Posterior temporalis	4,9	1,5	2,2	5,1	1,9
*K*_*Ni*_ [×10^3^ N/m]						
Non-Working	Masseter	78,6	24,1	32,8	82,7	31,3
	Medial pterygoid	106,5	33,4	45,0	113,2	42,6
	Temporalis	9,4	2,8	4,1	9,8	3,6
	Anterior temporalis	7,4	2,3	3,2	7,7	2,9
	Middle temporalis	4,5	1,4	1,9	4,7	1,7
	Posterior temporalis	3,6	1,1	1,5	3,7	1,4

**TABLE 5:** Intrinsic strength values (*k*_*Wi*_ *, k*_*Ni*_) for selected muscles in relation to food.

Side	Muscle	*PCSA*_*j*_ /^1^ [×10^−4^ m^2^]	Chocolate (d)	Chocolate bar (b)	Apple (a)	Carrots (c)	Sausage (s)
*k*_*Wi*_ [×10^4^ N/m^2^]							
Working	Masseter	6.80	34.3	22.0	16.5	40.3	18.7
	Medial pterygoid	4.37	47.9	30.8	23.0	56.0	26.0
	Temporalis	8.23	5.6	3.6	2.8	6.5	3.0
*k*_*Ni*_ [×10^4^ N/m^2^]							
Non-Working	Masseter	6.80	29.5	18.8	14.1	34.6	16.0
	Medial pterygoid	4.37	41.0	26.4	19.7	47.9	22.3
	Temporalis	8.23	4.8	3.0	2.4	5.6	2.5

/^1^ Langenbach and Hannam, 1999.

In the general case, the total contraction of the muscle (*Δq*_*C*_), the contraction corresponding to the maximum force (*Δq*_*H*_) and the muscle contraction (*Δq*_*E*_) were determined from Eqs 1–3. During the calculations, the equations were modified in such a way that the above parameters could be determined on selected muscles depending on the food, separately for the working and non-working sides.

$$\Delta q_{C}=\left|q\left(t\right)-q\left(0\right)\right|$$ (1)

$$\Delta q_{H}=\left|q\left(t_{H}\right)-q\left(0\right)\right|$$ (2)

$$\Delta q_{E}=\left|q\left(t_{E}\right)-q\left(0\right)\right|$$ (3)

where:

*q*(0)—initial muscle length for time *t = 0s*—open mouth,

*q*(*t*)—muscle end length for time *t*—closed mouth,

*q*(*t*_*H*_)—muscle length for time *t*_*H*_, corresponding to the position of the mandible in which the muscle force reaches its maximum value (*F*_*H*_),

*q*(*t*_*E*_)—muscle length determined for time *t*_*E*_ from the interval *0÷t*,

### 3.1 Muscle force patterns

Preliminary analysis indicates significant differences between patterns, force values and contractions. The variation in muscle behaviour is not surprising, as the diversity of characteristics (Figure 1) generates individual conditions for the masticatory system (Mioche et al., 1999), adapted to functional requirements, depending on the input data (Fitts et al., 1991).

**FIGURE 7:**
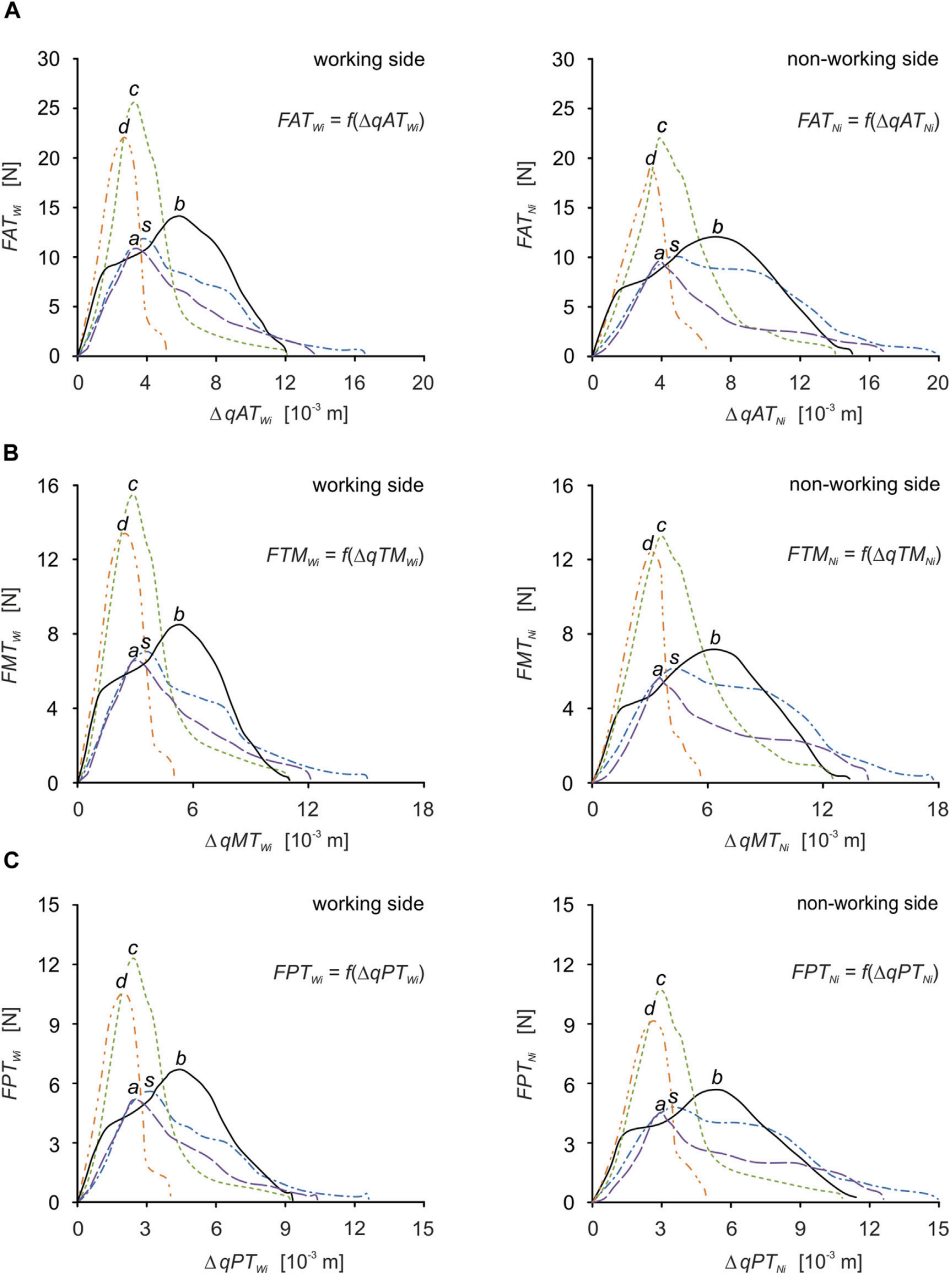
Muscle force patterns as a function of muscle force vs. muscle contraction for: **(A)** the anterior temporalis [*FAT*_*Wi*_ *= f*(*ΔqAT*_*Wi*_), *FAT*_*Ni*_ *= f*(*ΔqAT*_*Ni*_)], **(B)** the middle temporalis [*FMT*_*Wi*_ *= f*(*ΔqMT*_*Wi*_), *FMT*_*Ni*_ *= f*(*ΔqMT*_*Ni*_)] and **(C)** the posterior temporalis [*FPT*_*Wi*_ *= f*(*ΔqPT*_*Wi*_), *FPT*_*Ni*_ *= f*(*ΔqPT*_*Ni*_)] during unilateral food chewing (*i*).

For efficient interpretation of the results, muscle patterns were prepared separately for each muscle (Figure 7) and for the components of the temporalis muscle (Figure 5), and separately for the working and non-working sides. Thanks to this, there are 5 muscle patterns on each graph, depending on the food, while the data are given in Tables 2, 3 enable the determination of additional mechanical parameters characterising the muscle functioning.

### 3.2 Stiffness of the muscle

In the general case, the stiffness (*K*) is determined from the elementary Eq. 4, in which the force (*F*) and displacement (*x*) are directly proportional to each other.

$$K=F\;/\;x\;\left[N/m\right]$$ (4)

Eq. 4 is linear, so its use is limited to problems with a linear relationship between *F* and *x*. This means that the stiffness determined for a range of load (0*÷F*) or displacement (0*÷x*) has a constant value.

The above equation can be used for issues of a non-linear nature, but then the stiffness is only determined for a specific value of *F* or *x*. Therefore, the value of stiffness has the characteristics of a comparative parameter based on which it is possible to assess the influence of various factors on the functioning of the muscular system, e.g., 1) different ways of biting, 2) the geometry of the masticatory system resulting from individual characteristics and pathological conditions, 3) foods, 4) the state of dentition and 5) changes resulting from surgical procedures (Pachnicz and Stróżyk, 2021).

Based on the considerations above, this paper is limited only to the determination of muscle stiffness (Eq. 4) for the maximum muscle forces (Table 2, 3) for the working (*K*_*Wi*_) and non-working (*K*_*Ni*_) sides, respectively.

Of course, Eq. 4 was adjusted so that stiffness could be determined for selected muscles, i.e., *F*→*F*_*H*_ and x→*ΔqH*.

### 3.3 Intrinsic strength

Based on publications (Koolstra et al., 1988; Korioth and Hannam, 1990; May et al., 2001; Pruim et al., 1980) it can be stated that intrinsic strength (*k*), is used to determine maximum muscle force (*F*_*H*_) based on Eq. 5 and the relationship between muscle force (*F*) and EMG measurements according to Eq. 6.

$$F_{H}=k\times PCSA$$ (5)

$$F=k\times PCSA\times EMG_{F}$$ (6)

where: (*EMG*_*F*_)—scale factor

To calculate intrinsic strength, Eq. 5 was used, which was adjusted for the selected muscles depending on the selected foods, for the working (*k*_*Wi*_) and non-working (*k*_*Ni*_) sides, respectively—Table 5. To determine intrinsic strength, the data given in Table 1–3 were used and it was assumed that the *PCSA* values on the working and non-working sides are the same.

The values of the temporalis muscle components are not included in Table 5 because their values are identical to those of the principal vector.

## 4 Discussion

In the work presented here, we used our model of unilateral chewing (Figure 2) developed based on: 1) the anatomical structure of the masticatory system (Synbone skull 8500 and mandible 8596), 2) food patterns Figures 1, 3 chewing paths Figure 3A.

Based on the assumptions made, dynamic patterns were determined for the mandibular elevator muscles in the form of muscle forces and corresponding contractions. Furthermore, the results obtained allowed the calculation of stiffness of the muscle and intrinsic strength. All values of the above parameters were determined in relation to the food and the working and non-working sides for a single chewing cycle.

### 4.1 Limitations of the model

The model proposed and the assumptions made are a compromise between a correctly anatomical structure of the masticatory system and the physiological mechanism of mastication (act of chewing) and the possibility of reproducing the conditions (parameters) necessary to reproduce physiologically correct unilateral chewing.

Despite the use of simplifications (e.g., same chewing velocity, the muscle is represented by a single principal vector, no friction coefficients, no consideration of the muscle damping coefficient, use of a single geometrical model of the mandible, mechanically correct but basic in terms of the anatomical structure temporomandibular joint), the model meets the basic requirements to carry out calculations in accordance with the principles of solid mechanics and function of the masticatory system.

Since the model does not take into account all the parameters responsible for the correct course of the chewing process, it must be assumed that the results of the calculations show how the mandibular elevator muscles may function during the first unilateral chewing cycle.

### 4.2 Dynamic patterns of muscle forces

Similar to the authors’ previous work (Stróżyk and Bałchanowski 2016; Stróżyk and Bałchanowski, 2018; Stróżyk and Bałchanowski, 2020) and other publications (Agrawal et al., 1998; Hiiemae et al., 1996; Koolstra, 2002; Mathevon et al., 1995; Shimada et al., 2012) and the present study, it has been shown that mechanical [*F*_*i*_ *= f*(Δ*h*_*i*_)] and geometric [height (*h*_*i*_)] food parameters have a significant influence on the function of the masticatory system, especially the muscular system.

Based on the elementary comparative analysis of the food characteristics (Figure 1) with the muscle force patterns (Figures 6, 7), it can be shown that there is a high similarity between them related to the course of the graphs. Such a relationship occurs in the so-called simple mechanisms (Stróżyk and Bałchanowski, 2016; Stróżyk and Bałchanowski, 2018; Stróżyk and Bałchanowski, 2020) characterised by the fact that the response of the system is in the form of displacements and enforcing forces (muscle forces) is correlated with the force (resistance force posed by food). The correlation between the above-mentioned parameters is manifested, among other things, by their values increasing at the same rate and reaching maximum values at the same time. Of course, the above statement is fulfilled for the kinematically correct movement of the masticatory system.

Furthermore, it can also be seen that the patterns of the same muscle on the working and non-working sides have similar characteristics but different values (Tables 2, 3). This is due to the asymmetrical alignment of the mandible concerning the sagittal plane, which is a consequence of including the chewing path.

In addition, significant differences can be observed, irrespective of the muscle, between the values of maximal muscle forces and maximal contractions, depending on the food. However, the comparison of the values of the contractions corresponding to the maximum forces shows that the differences are not as explicit as for the muscle forces (Tables 2, 3).

#### 4.2.1 Maximum muscle force

The results indicate that not only muscle force patterns but also maximum muscle forces are dependent on food (mechanical parameters). Moreover, analysing their values (Tables 2, 3) it was observed that on the working side, they are higher than on the non-working side. This result proves the correctness of the obtained results and compliance with the elementary static principle, from which it follows that the resultant of external forces (occlusion force) is located closer to the components (muscular forces) with higher values.

A detailed analysis taking into account the values of the maximum muscle forces shows that the forces on the non-working side are on average 14% lower than those on the working side, irrespective of the muscle (the masseter, medial pterygoid and temporalis muscles), the division of the temporalis muscle (Figure 5) and the food (Table 1). The difference is not large, but it balances the non-working and the working side, allowing a stable mandible elevation during unilateral chewing for the given boundary conditions.

The knowledge of the maximum muscle forces (*F*_*HWi*_ and *F*_*HNi*_) and the maximum occlusion forces (*F*_*i*_ *,*_*max*_) made it possible to show that there is a correlation between them, which can be presented in the form of their ratio (Table 6), corresponding for the working (*R*_*FW*_) and non-working (*R*_*FN*_) sides. Based on the data (Figure 1; Tables 2, 3) *R*_*FW*_ and *R*_*FN*_ can be determined considering: (1) the principal vectors of the masseter, medial pterygoid and temporalis muscles (case I) and/or (2) the principal vectors of the masseter muscle and the medial pterygoid muscle with simultaneous consideration of the division of the temporalis muscle into three parts (case II)—(Figure 5). The results given in Table 6 indicate that the values of *R*_*FW*_ and *R*_*FN*_ are constant for the muscles and components of the primary vector of the temporalis muscle, of food, but different for the working and non-working sides.

**TABLE 6:** The value of proportionality coefficients *R*_*FW*_ and *R*_*FN*_ for selected muscles, depending on cases I and II.

Side	Muscle	Case I	Case II
*R*_*FW*_			
Working	Masseter	1.215	
	Medial pterygoid	1.089	
	Temporalis	0.239	–
	Anterior temporalis	–	0.115
	Middle temporalis	–	0.069
	Posterior temporalis	–	0.055
***R***_***FN***_			
Non-Working	Masseter	1.042	
	Medial pterygoid	0.934	
	Temporalis	0.204	–
	Anterior temporalis	–	0.098
	Middle temporalis	–	0.059
	Posterior temporalis	–	0.047

The relationship between maximum muscle force and maximum occlusion force can also be represented by a linear relationship (Eqs 7, 8) in which the proportionality coefficient is *R*_*FW*_ and *R*_*FN*_.

$$F_{HWi}=R_{FW}\times F_{i,\max}$$ (7)

$$F_{HNi}=R_{FN}\times F_{i,\max}$$ (8)

In the literature, it is possible to find publications in which authors model bilateral and unilateral chewing, among others (Choi et al., 2005; Pinheiro and Alves, 2015; Jahadakbar et al., 2016; Lee et al., 2017; Luo et al., 2017; Marková and Gallo, 2016; Orassi et al., 2021; Pachnicz and Stróżyk, 2021; Reina et al., 2007); however, these are models corresponding to static occlusion and the muscle force values used are based, primarily, on the data reported in (Korioth et al., 1992; Korioth and Hannam, 1994) and in (Ferrario et al., 2004).

Despite the use of static models in the above-mentioned publications, it was decided to relate the obtained maximum values of muscle forces to those works in which the authors take into account the unilateral loading of the mandible—Table 7. Taking into account the data provided by (Pinheiro and Alves, 2015), it follows that the values of forces in the muscles differ significantly from those reported in Tables 2, 3. According to the authors of the above-mentioned publication, the main role in generating occlusal force is played by the temporalis muscle, which muscular force is greater than that of the masseter muscle and the medial pterygoid muscle, both on the working and non-working sides. On the basis of the results obtained in Tables 2, 3, it follows that during unilateral chewing the muscle forces generated by the masseter muscle and the medial pterygoid muscle are significantly greater than the values determined for the temporalis muscle. A similar regularity regarding the values of muscle forces can be found in the work of (Korioth et al., 1992).

**TABLE 7:** Values of muscle forces corresponding to unilateral chewing.

Muscle		Pinheiro and Alves (2015)		Reina et al. (2007)		Korioth et al. (1992)	
		Right	Left	Right	Left	Right	Left
Superficial masseter	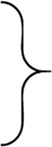	234.1	195.1	106.6	38.1	137.1	114.2
Deep masseter				45.7	16.3	58.7	49.0
Medial pterygoid		191.0	136.4	169	82.2	146.2	104.4
Lateral pterygoid		26.1	56.5	33.5	23.9	20.1	43.5
Anterior temporalis	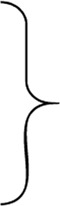	263.6	221.4	102.7	80.6	115.3	91.6
Middle temporalis				57.4	50.7	63.1	64.1
Posterior temporalis				40.8	40.8	44.6	29.5

Based on the muscle force values reported by (Reina et al., 2007), it appears that the medial pterygoid muscle is the dominant muscle. The analysis performed indicates that on the working side, the medial pterygoid muscle has the highest values, while the superficial the masseter muscle and the temporalis muscle have similar values. On the non-working side, the medial pterygoid muscle and the posterior temporalis muscle have similar values, while the masseter muscle has a value ∼2 times lower than them. In contrast, the results are shown in Tables 2, 3 indicate that regardless of the product and side, the masseter muscle has the highest value, and the temporalis muscle has the lowest value. The muscle force values of the medial pterygoid muscle are only 10% less than those of the masseter muscle.

Furthermore, in the works (Korioth et al., 1992; Reina et al., 2007), the temporalis muscle is decomposed into 3 components (Table 7), between which there is the same relationship as shown in Table 3, i.e., the anterior temporalis muscle has the highest value of muscle force, the middle temporalis muscle intermediate and the posterior temporalis muscle the lowest, regardless of the side.

#### 4.2.2 Muscle contraction

On the basis of the data given in Tables 2, 3, it can be shown that for the working side, the total contraction of the mandibular elevator muscles is 17% lower on average than on the non-working side.

Analysis of the results also showed that total contraction was dependent on the initial food height (Table 1), i.e., the distance between a pair of corresponding molars.

Detailed analysis showed that the height of the food influences: 1) the size of the chewing path (mainly height, but also width (Figure 3) and 2) muscle contractions (Tables 2, 3). The relationship between food height and chewing path height in the sagittal plane can be described by Tales’ theorem. On the other hand, the relationship between the height of the food (*h*_*i*_) and the total muscle contraction (*Δq*_*Ci*_) can be determined: 1) by solving tasks in which the movable part (mandible) is suspended on a tendon (muscle) and is simultaneously supported at a point corresponding to the *TMJ* or 2) using a parameter that is the ratio (ratio) of their values (*Δq*_*Ci*_/*h*_*i*_). The ratio was determined separately for the working side (*R*_*qW*_) and the non-working side (*R*_*qN*_). The results of the calculations (Table 8) showed that the values of *R*_*qW*_ and *R*_*qN*_ are: 1) constant for each muscle and 2) dependent on the considered side, i.e., working or non-working side. Considering the temporalis muscle (Figure 5), the values of *R*_*qW*_ and *R*_*qN*_ (Table 8) can be presented for 2 cases: 1) without considering the division of the temporalis muscle (case I) and 2) with considering the division of the temporalis muscle into three parts (case II) (Figure 5).

**TABLE 8:** Values of proportionality coefficients *R*_*qW*_ and *R*_*qN*_ for selected muscles, depending on cases I and II.

Side	Muscle		Case I		Case II	
*R*_*qW*_						
Working		Masseter		0.46		
		Medial pterygoid		0.29		
		Temporalis		0.74		–
		Anterior temporalis		–		0.60
		Middle temporalis		–		0.53
		Posterior temporalis		–		0.46
***R***_***qN***_						
Non-Working	Masseter		0.55			
	Medial pterygoid		0.36			
	Temporalis		0.90		–	
	Anterior temporalis		–		0.73	
	Middle temporalis		–		0.64	
	Posterior temporalis		–		0.55	

The relationship between (*Δq*_*Ci*_) and (*h*_*i*_) can be represented by the linear relationship (Eqs 9, 10) in which the proportionality coefficient is *R*_*qW*_ and *R*_*qN*_.

$$\Delta q_{CWi}=R_{qW}\times h_{i}$$ (9)

$$\Delta q_{CNi}=R_{qN}\times h_{i}$$ (10)

Based on the results presented, it can be assumed that the muscle contraction during unilateral chewing, with the dimensions of the masticatory system fixed, depends on the height of the food bite. Since the height (*h*_*i*_) changes during biting, therefore, the contraction can be written in the form of a function *Δq*_*E*_ = *f*(*Δh*_*i*_) - the muscle contraction (*Δq*_*E*_) vs. the height of food specimen (*Δh*_*i*_). Moreover, the above parameters are time-dependent, i.e., *Δq*_*E*_(*t*) and *Δh*_*i*_(*t*). The above conclusion can also be applied to symmetric incisal biting of foodstuffs.

There was no clear relationship between maximum force and corresponding contraction (Tables 2, 3).

### 4.3 Stiffness of the muscle

As with the other parameters, the stiffness of the muscle (*K*) is also strongly dependent on: 1) the mechanical properties of the food (texture), 2) the muscle analysed and 3) the side analysed, i.e., working and/or non-working.

Preliminary analysis of the results indicates that similar values of stiffness of the muscle (Table 4) can be obtained for products different in texture, characteristics (Figure 1) and method of manufacture. The results given in Table 4 indicate that with respect to *K* the selected products can be divided into two groups, i.e., 1) chocolate and carrot and 2) bar, apple and sausage. It can also be observed that the *K* in the first group is, on average 2.7 times higher than that of the second group, irrespective of the type of muscle and the working and non-working side. Furthermore, analysing the *K* values in relation to the side, it can be seen that the stiffness of the muscles is lower under the non-working side (*K*_*Ni*_), compared to the working side (*K*_*Wi*_), by an average of 29%.

Analysis of the results given in Tables 2–4 indicates that the greater the stiffness of the muscle, the greater the maximum muscle force.

It should be added that the determined patterns of muscle forces (Figures 6, 7) enable the determination of the non-linear stiffness (*K*_*i*_) according to the general Eq. 11. However, first, with the help of an appropriate tool (mathematical program), an appropriate mathematical equation should be fitted to the data (patterns), i.e., *F*_*Ei*_ = *f*(*ΔqEi*) or *F*_*Ei*_ = *F*_*Ei*_(*ΔqEi*) (muscle force vs. muscle contraction).

$$K_{i}=dF_{Ei}\;/\;d\left(\Delta qi\right)$$ (11)

where: *dF*_*Ei*_—muscle force gains in relation to food and side of the mandible, *d*(*Δqi*)—increases in muscle contraction according to food and side of the mandible

The resulting *K*_*Wi*_ and *K*_*Ni*_ values can be used in numerical calculations where the muscle is modelled as a spring only (Antic et al., 2015; Pachnicz and Stróżyk, 2021). If the calculations involve static loading, it is sufficient to provide a single value corresponding to a given bite force or muscle force, depending on the target. In dynamic calculations, the non-linear function of the muscle must be taken into account (Figure 6), so the stiffness must be entered as a function according to the requirements of the programme. On the other hand, an accurate representation of the performance characteristics of the muscle requires the use of complex models, e.g., 1) Maxwell model, 2) Voigth model, 3) Kelvin model, and 4) Hill model, in which additional parameters regarding elements such as spring elements, damping elements, contractile element and parallel elastic element (elements describing the passive elastic properties of the muscle fibres) must be taken into account (Romero and Alonso, 2016). It is also possible to use the model proposed by Röhrle and Pullan (2007), in which the muscle is a viscoelastic material.

### 4.4 Intrinsic strength

Analysis of the data given in Table 5 has shown that the values of intrinsic strength (*k*): 1) depend on the food, above all on its mechanical properties, 2) depend on the side analysed, i.e., working and/or non-working, 3) regardless of the side and food considered, are the highest for the temporalis muscle and the lowest for the medial pterygoid muscle. However, for the masseter muscle they are smaller than the medial pterygoid muscle by an average of 28%, and 4) they are identical both for the main vector of the temporalis muscle and its components (this results from an assumption related to the decomposition of the main vector into its components (Figure 5).

In the available literature (in recent years), there are few publications concerning the determination of the *k*-parameter, especially in relation to physiological forces. An interesting publication on the *k*-parameter, despite the passage of more than 40 years, is the paper by Pruim et al., 1980. In the above-mentioned paper, the authors provide *k* values determined during experimental studies (*in vivo*) but during bilateral static occlusion at different levels of occlusion force. Moreover, the authors assume the same *k* value for all muscles, which makes it difficult to compare the results. However, taking the values obtained for chocolate as a reference (lowest height and maximum occlusion force reached in the mandibular position close to the close-up of the teeth) and considering the temporalis muscle (lowest *k*), it was decided to compare the results given by Pruim et al., 1980 with the values presented in Table 5. Analysis of the value of the *k* parameter showed that during unilateral chewing, the temporalis muscle has significantly lower average values (more than 20 times lower than bilateral chewing). Considering the masseter muscle and the medial pterygoid muscle, it turns out that the mean values are also smaller, respectively: 4-fold and 3-fold.

Taking into account some of the oldest data concerning the parameter *k* given in the works by Fick et al. (1904)-1 × 10^6^ N/m^2^, Morris (1948)-0.9 × 10^6^ N/m^2^, Ikai and Fukunaga (1968)-0.7 × 10^6^ N/m^2^ it follows that a value close to the data given in Table 5 is 0.4 × 10^6^ N/m^2^ determined by Hettinger. (2017). A similar value in the 1980s was given by Weijs and Hillen (1985) i.e., *k = 0.*37 × 10^6^ N/m^2^, which is widely used by many authors (Hattori et al., 2003; Koolstra et al., 1988; Koolstra and Eijden, 1992; Korioth and Hannam, 1990; Langenbach and Hannam, 1999; Peck et al., 2000; Zheng et al., 2019). Analysis of the aforementioned papers indicates that the authors use only one value of *k =* 0.37 × 10^6^ N/m^2^ or 0.4 × 10^6^ N/m^2^, although the paper (Slager et al., 1997) used *k* = 0.35 × 10^6^ N/m^2^. Furthermore, the authors used one value of *k* for all muscles. However, according to the data given in Table 5, it follows that the values of *k* are different and depend on the food product. This indicates that the mechanical parameters of the product also have a significant influence on the values of the *k* parameter. The same observations but concerning chewing, can be found in (Stróżyk and Bałchanowski, 2018).

It should also be added that the equations (Eqs 5, 6) from which the value of *k* was determined are linear and restricted to a point only, i.e., *F*_*H*_.

It is interesting to note that *k* is very strongly dependent on PCSA, i.e., for the same muscle force but different values of PCSA, different values of *k* can be obtained, and each will bear the hallmarks (or give the impression) of a correct value.

It is also worth noting that there is an analogy between intrinsic strength (*k*) and the classical definition of strain (σ), i.e., the same formula and the same physical unit in the SI system. Considering the general function *σ = f*(*ε*) (tension vs. strain), which is used for the mathematical notation of the characteristics obtained in the classical compression test, it is possible, on its basis, to propose an elementary function for the parameter *k*, i.e., *k = f*(*ε*), where*:ε = Δq*_*E*_ */q*(0). On the basis of the above function, it can be shown that *k* is not a constant value during muscle contraction and changes with the change in load similarly to σ. The equation *k = f*(*ε*), does not change the physical meaning of intrinsic strength but extends its applications to the full range of changes in muscle force values, not only the maximum values.

## 5 Conclusion

The present work shows that the use of a hybrid model based on numerical simulation and experimental studies can provide an advanced (promising) tool for modelling complex processes such as the act of chewing. This paper also shows that muscle forces and mechanical parameters of food can be represented as functions, *F*_*i*_ = *f*(*q*_*i*_) and *F*_*i*_ = *f*(Δ*h*_*i*_), respectively. In addition, the advantage of the presented model is that it allows rapid modification of geometric parameters and loading functions tailored to individual patient characteristics. The results of such calculations will allow for better fabrication and fitting of implants or prosthetic components to the patient.

The determined muscle or food force patterns can also be used in numerical simulations based on deformable body mechanics. Such simulations will make it possible to determine stress, strain and displacement at selected points in the mandible, maxilla, teeth and temporomandibular joint.

The conclusions of the present study confirm and complement theories about the mechanisms of the masticatory system during chewing. On the other hand, the computational technique used, using inverse kinematics and dynamics analysis and the application of food patterns, made it possible to understand the influence of mechanical food parameters on muscle force patterns.

## Data Availability

The raw data supporting the conclusion of this article will be made available by the authors, without undue reservation.
